# Supplementary material for: Long term fMRI adaptation depends on adapter response in face-selective cortex
Source: Commun Biol. 2021 Jun 10;4:712. doi: 10.1038/s42003-021-02235-6 (PMC8192765; doi:10.1038/s42003-021-02235-6)
Supplement: Supplementary file 2 — Supplementary Information [file 42003_2021_2235_MOESM2_ESM.pdf]

Supplementary Information

Supplementary method

In addition to our main experiment, we conducted an eye tracker experiment outside the scanner using the same procedures (fixation at the fixation cross was instructed but not enforced). This was performed in an independent sample of 20 participants that were demographically matched to the fMRI sample at group level [6 males (30 %); mean age ± SD = 37 ± 20 years, range 21-67]. Independent Sample t-test showed that no significant differences were detected for age ($p=.538$). Chi-Square test showed no significant differences for sex ($p=.604$).

Eye movement data was collected at a sampling rate of 120 Hz using the Tobii eye tracker TX300[1] and Tobii Studio 3.4.7. A five-point fixation position calibration was performed prior to the experiment. We applied default settings, including the Tobii fixation filter, with a velocity threshold of 0,84 pixels/ms (35 pixels) and a distant threshold (distance between two consecutive fixations) of 35 pixels (default). A detailed description of the Tobii fixation can be found in the Tobii Studio user manual (https://www.tobiipro.com/siteassets/tobii-pro/user-manuals/tobii-pro-studio-user-manual.pdf). Before data acquisition, we created 3 Areas of Interest (AOI), corresponding to the mouth, nose, and eyes within Tobii Studio.

Subsequently, we created group level heatmaps during presentation of 3 conditions: fixation, faces, and houses, using default eye tracker settings (https://www.tobiipro.com/siteassets/tobii-pro/user-manuals/tobii-pro-studio-user-manual.pdf).

The fixation cross was positioned around the center of the stimuli, which for faces consisted of the midpoint of the virtual line connecting the nasion and nasal septum (Fig. S1a). The results show that during the presentation of the fixation cross, the vast majority of fixations cover the area where the fixation cross was shown for all participants (Fig. S1b and S1c). Overall, the results reveal task-compliant fixation on the fixation cross and a majority of similar fixation positions during presentation of the face and house stimuli.

Finally, we analysed the difference in number of fixations between the adapter and test stimuli, within each heatmap. Mann Whitney U test revealed no significant difference in fixations over time for both categories (face, house; all p's>.280).

Supplementary Figure and Figure Legend

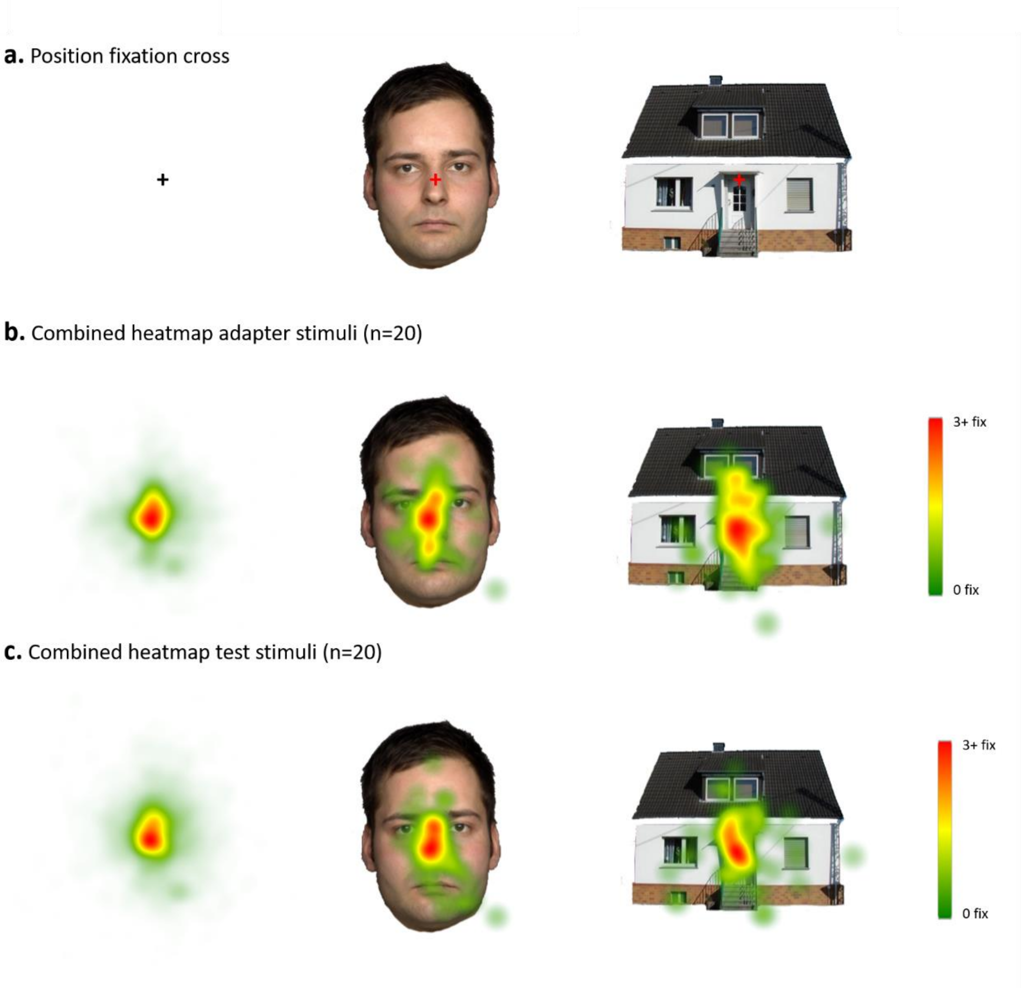

Supplementary Figure 1: Position fixation cross and heatmaps.

a. Red fixation cross illustrates where the fixation cross was positioned relative to the stimuli. For the face stimuli the fixation cross was positioned around the midpoint of the virtual line connecting the nasion and nasal septum. **b.** Group level (n=20) heatmaps of adapter stimuli, during presentation of 3 conditions: fixation, faces and houses. **c**. Group level (n=20) heatmaps of test stimuli, during presentation of 3 conditions: fixation, faces and houses.
