# Supplementary material for: Long term fMRI adaptation depends on adapter response in face-selective cortex
Source: Commun Biol. 2021 Jun 10;4:712. doi: 10.1038/s42003-021-02235-6 (PMC8192765; doi:10.1038/s42003-021-02235-6)
Supplement: Supplementary file 3 — Description of Supplementary Files [file 42003_2021_2235_MOESM3_ESM.pdf]

Description of Additional Supplementary Files

File name: Supplementary Data 1

Description: Source data underlying the graphs (Fig. 2-6).
