# Supplementary material for: Long term fMRI adaptation depends on adapter response in face-selective cortex
Source: Commun Biol. 2021 Jun 10;4:712. doi: 10.1038/s42003-021-02235-6 (PMC8192765; doi:10.1038/s42003-021-02235-6)
Supplement: Supplementary file 5 — Reporting Summary [file 42003_2021_2235_MOESM5_ESM.pdf]

nature research

Corresponding author(s): Jan Van den Stock

Last updated by author(s): 19/04/21

Reporting Summary

Nature Research wishes to improve the reproducibility of the work that we publish. This form provides structure for consistency and transparency in reporting. For further information on Nature Research policies, see our Editorial Policies and the Editorial Policy Checklist.

Statistics

For all statistical analyses, confirm that the following items are present in the figure legend, table legend, main text, or Methods section.

n/a	Confirmed	
☐	☒	The exact sample size (*n*) for each experimental group/condition, given as a discrete number and unit of measurement
☐	☒	A statement on whether measurements were taken from distinct samples or whether the same sample was measured repeatedly
☐	☒	The statistical test(s) used AND whether they are one- or two-sided<br>*Only common tests should be described solely by name; describe more complex techniques in the Methods section.*
☐	☒	A description of all covariates tested
☐	☒	A description of any assumptions or corrections, such as tests of normality and adjustment for multiple comparisons
☐	☒	A full description of the statistical parameters including central tendency (e.g. means) or other basic estimates (e.g. regression coefficient) AND variation (e.g. standard deviation) or associated estimates of uncertainty (e.g. confidence intervals)
☐	☒	For null hypothesis testing, the test statistic (e.g. *F*, *t*, *r*) with confidence intervals, effect sizes, degrees of freedom and *P* value noted<br>*Give P values as exact values whenever suitable.*
☒	☐	For Bayesian analysis, information on the choice of priors and Markov chain Monte Carlo settings
☒	☐	For hierarchical and complex designs, identification of the appropriate level for tests and full reporting of outcomes
☐	☒	Estimates of effect sizes (e.g. Cohen's *d*, Pearson's *r*), indicating how they were calculated

Our web collection on statistics for biologists contains articles on many of the points above.

Software and code

Policy information about availability of computer code

Data collection	No software was used
Data analysis	- Imaging data were analyzed using BrainVoyager 21.4<br>Goebel, R. BrainVoyager — Past, present, future. NeuroImage 62, 748–756 (2012).<br>- IBM SPSS Statistics 26 for statistical analysis<br>IBM Corp. (2017). IBM SPSS Statistics for Windows, Version 25.0. Armonk, NY: IBM Corp.

For manuscripts utilizing custom algorithms or software that are central to the research but not yet described in published literature, software must be made available to editors and reviewers. We strongly encourage code deposition in a community repository (e.g. GitHub). See the Nature Research guidelines for submitting code & software for further information.

Data

Policy information about availability of data

All manuscripts must include a data availability statement. This statement should provide the following information, where applicable:
- Accession codes, unique identifiers, or web links for publicly available datasets
- A list of figures that have associated raw data
- A description of any restrictions on data availability

The data supporting the findings of this study are available within the paper and its Supplementary Information files. Source data are provided with this paper.

Field-specific reporting

Please select the one below that is the best fit for your research. If you are not sure, read the appropriate sections before making your selection.

☐ Life sciences ☒ Behavioural & social sciences ☐ Ecological, evolutionary & environmental sciences

For a reference copy of the document with all sections, see nature.com/documents/nr-reporting-summary-flat.pdf

Behavioural & social sciences study design

All studies must disclose on these points even when the disclosure is negative.

Study description	Quantitative methods
Research sample	56 healthy subjects participated in our study. The final sample for the main analyses consisted of fifty-four subjects [13 males (24 %); mean age ± SD = 34 ± 11 years, range 21-64].<br><br>(An additional 20 participants that were demographically matched to the fMRI sample at group level [6 males (30 %); mean age ± SD = 37 ± 20 years, range 21-67])
Sampling strategy	Subjects were recruited via advertisements for participation in an fMRI memory experiment.
Data collection	Pictures were projected onto a screen and were viewed through a mirror mounted on the head coil, minimizing head movements. Brain imaging was performed on a 3T Siemens Achieva scanner, using a 32-channel head coil. Responses were recorded via an MR-compatible keypad (MRI Devices, Waukesha, WI), positioned on the right side of the participant's abdomen. A desktop workstation running PRESENTATION® 19.0 (Neurobehavioral Systems, San Francisco, CA) controlled stimulus presentation and response registration.<br>Only the researcher and participant were present.<br><br>(- Tobii eye tracker TX300 and Tobii Studio 3.4.7<br>Tobii Technology AB. http://www.tobii.se. (Sweden))
Timing	First inclusion fMRI: 18/06/2013<br>Last inclusion fMRI: 25/01/2019
Data exclusions	One participant was excluded due to technical failure during fMRI acquisition and one participant was excluded due to indication of pathology.
Non-participation	No drop-out
Randomization	Participants were not allocated into experimental groups

Reporting for specific materials, systems and methods

We require information from authors about some types of materials, experimental systems and methods used in many studies. Here, indicate whether each material, system or method listed is relevant to your study. If you are not sure if a list item applies to your research, read the appropriate section before selecting a response.

Materials & experimental systems

n/a	Involved in the study	
☒	☐	Antibodies
☒	☐	Eukaryotic cell lines
☒	☐	Palaeontology and archaeology
☒	☐	Animals and other organisms
☐	☒	Human research participants
☒	☐	Clinical data
☒	☐	Dual use research of concern

Methods

n/a	Involved in the study	
☒	☐	ChIP-seq
☒	☐	Flow cytometry
☐	☒	MRI-based neuroimaging

Human research participants

Policy information about studies involving human research participants

Population characteristics	See above
Recruitment	Subjects were recruited via advertisements for participation in an fMRI memory experiment.
Ethics oversight	The study was approved by the Ethical Committee of University Hospitals Leuven. All subjects gave written informed consent in accordance with the Declaration of Helsinki.

Note that full information on the approval of the study protocol must also be provided in the manuscript.

Magnetic resonance imaging

Experimental design

Design type	Event-related design
Design specifications	The experiment consisted of an encoding phase, an immediate recognition (IR) phase, and a delayed recognition phase (DR). The encoding phase consisted of two blocks, each in a separate run of 8.48 minutes. In each run, 160 stimuli (80 houses and 80 faces) were pseudo-randomly presented for 1500ms against a white background, separated by a 1000ms ISI during which a black fixation cross was presented. In addition, 40 null-events (1500ms) were included during which only the fixation cross was displayed.<br>The IR phase directly followed the encoding phase. In the IR phase, the stimuli consisted of the 160 stimuli from the encoding phase intermixed with an additional 80 distractors: 40 houses and 40 faces. Sixty null-events were interspersed. The procedure in the IR phase consisted of presentation of a stimulus (1500ms) followed by a response screen (3000ms).<br>The 300 trials (160 targets + 80 distracters + 60 null-events) were equally divided over 4 runs of 7.48 minutes each and each comprising 75 trials: 30 houses (20 from the encoding phase), 30 faces (20 from the encoding phase), and 15 null-events.<br>The DR is conducted two days after the first session. The DR is identical to the IR, except for the stimulus presentation order and the distracter stimuli.
Behavioral performance measures	We recorded button press (answser). Behavioural results were analysed according to signal detection theory. R-Score Plus was used to calculate d’ for confidence rating designs. D’ was calculated.

Acquisition

Imaging type(s)	Structural and functional MRI
Field strength	3T
Sequence & imaging parameters	Brain imaging was performed on a 3T Siemens Achieva scanner, using a 32-channel head coil. Acquisition parameters for 45 participants consisted of a high-resolution T1-weighted<br>anatomical image (voxel size: 0.98 x 0.98 x 1.20 mm3) using a 3D turbo field echo sequence (TR:9.6 ms; TE:4.6 ms; matrix size:256 x 256; 182 slices); a T2*-weighted GE-EPI sequence with the following parameters: TR: 2000 ms; TE: 30 ms; matrix size: 80 x 78; FOV: 230 mm; flip angle: 90˚; slice thickness: 4 mm; no gap; axial slices: 38. For the other 9 participants, a similar high-resolution T1-weighted anatomical image was acquired (voxel size: 1.10 x 1.10 x 1.10 mm3) using a 3D turbo field echo sequence (TR:6.9 ms; TE:3.2 ms; matrix size:256 x 256; 208 slices) and a T2*-weighted GE-EPI sequence with the following parameters: TR: 2000 ms; TE: 30 ms; matrix size: 80 x 78; FOV: 230 mm; flip angle: 90˚; slice thickness: 4 mm; no gap; axial slices: 36. Scan acquisition setting was included as a nuisance variable in all brain imaging analyses.
Area of acquisition	Whole brain scan

Diffusion MRI ☐ Used ☒ Not used

Preprocessing

Preprocessing software	Imaging data were analyzed using BrainVoyager 21.4. Pre-processing of functional data consisted of slice scan time correction, temporal high-pass filtering to remove low-frequency drifts, realignment to the first image to compensate for head motion, and spatial smoothing with a Gaussian filter of 4mm FWHM.
Normalization	Functional data were co-registered with the anatomical images and normalized into Talairach coordinate space.
Normalization template	Functional data were co-registered with the anatomical images and normalized into Talairach coordinate space.
Noise and artifact removal	Imaging data were analyzed using BrainVoyager 21.4. Pre-processing of functional data consisted of slice scan time correction, temporal high-pass filtering to remove low-frequency drifts, realignment to the first image to compensate for head motion, and spatial smoothing with a Gaussian filter of 4mm FWHM.

Volume censoring | Imaging data were analyzed using BrainVoyager 21.4. Pre-processing of functional data consisted of slice scan time correction, temporal high-pass filtering to remove low-frequency drifts, realignment to the first image to compensate for head motion, and spatial smoothing with a Gaussian filter of 4mm FWHM.

Statistical modeling & inference

Model type and settings

At first level, the statistical analysis was based on the general linear model (GLM) with repetition (no, yes), category (face, house), and subsequent memory performance (Not, probably yes, and definitely yes) as factors. The 'definitely not' and 'probably not' conditions were pooled as 25 participants did not use both categories during their experiment in one of both categories. Null-events were modelled explicitly. At second level, a random effects GLM was performed.

Effect(s) tested

Linear Mixed Models (LMM) were estimated with the beta-values as dependent variable with repetition (2 levels: adapter and test), memory performance (3 levels: forgotten, probably remembered or definitely remembered), and repetition x memory performance as fixed effects using an unstructured variance-covariance matrix (based on a Likelihood test). These analyses were performed for every ROI separately. We applied Bonferroni-correction for multiple comparisons ('forgotten' vs 'probably remembered', 'forgotten' vs 'definitely remembered', and 'probably remembered' vs 'definitely remembered'). We only focused on within-performance category differences. In addition to the main effects of performance and repetition, we performed post-hoc analyses to study the interaction between performance and region and the interaction between repetition and region.

Specify type of analysis: ☐ Whole brain ☐ ROI-based ☒ Both

Anatomical location(s)

In order to define our category selective ROIs, we performed a whole brain analysis on the data, by contrasting all distractor face trials with all distractor house trials (q<.005, FDR-corrected). This revealed significant clusters comprising the topography of bilateral FFA and pSTS (faces>houses) on the one hand and PPA and RSC (houses>faces) on the other hand. Each of the 8 ROIs was subsequently defined at subject level by creating a 3mm-radius sphere around the subject-specific peak-voxel. The statistical threshold for ROI-definition at subject level was set at Pheight<.005. If no neural category-effect was found in a specific ROI, the subject was excluded for the analyses of this specific ROI.

Only ROI analyses were performed in category selective areas: Fusiform Face Area (FFA), posterior Superior Temporal Sulcus (pSTS), Parahippocampal Place Area (PPA), and RetroSplenial Cortex (RSC).

Statistic type for inference
(See Eklund et al. 2016)

Voxel-wise ROI analyses
In order to define our category selective ROIs, we performed a whole brain analysis on the data, by contrasting all distractor face trials with all distractor house trials (q<.005, FDR-corrected). This revealed significant clusters comprising the topography of bilateral FFA and pSTS (faces>houses) on the one hand and PPA and RSC (houses>faces) on the other hand. Each of the 8 ROIs was subsequently defined at subject level by creating a 3mm-radius sphere around the subject-specific peak-voxel. The statistical threshold for ROI-definition at subject level was set at Pheight<.005. If no neural category-effect was found in a specific ROI, the subject was excluded for the analyses of this specific ROI.

Only ROI analyses were performed in category selective areas: Fusiform Face Area (FFA), posterior Superior Temporal Sulcus (pSTS), Parahippocampal Place Area (PPA), and RetroSplenial Cortex (RSC).

Correction

FDR-corrected

Models & analysis

n/a	Involved in the study
☒	☐ Functional and/or effective connectivity
☒	☐ Graph analysis
☒	☐ Multivariate modeling or predictive analysis
